# Mortality and health survey, Walikale, Democratic Republic of the Congo, 2017: an example of the use of survey data for humanitarian program planning

**DOI:** 10.1186/s13031-019-0232-y

**Published:** 2019-11-21

**Authors:** Eve Robinson, Vittoria Crispino, Adelaide Ouabo, Francklin Brice Soung Iballa, Ronald Kremer, María Eugenia Serbassi, Marit van Lenthe, Antonio Isidro Carrion Martin

**Affiliations:** 1 European Programme for Intervention Epidemiology Training, European Centre for Disease Prevention and Control, Stockholm, Sweden; 2 Médecins sans Frontières, London, United Kingdom; 3 Médecins sans Frontières, Goma, Democratic Republic of the Congo; 4 Médecins sans Frontières, Amsterdam, the Netherlands

**Keywords:** Democratic Republic of the Congo, Health surveys, Morbidity, Mortality, Vaccination coverage, Mosquitoe bednets

## Abstract

**Background:**

During humanitarian crises, health information systems are often lacking and surveys are a valuable tool to assess the health needs of affected populations. In 2013, a mortality and health survey undertaken by Médecins Sans Frontières (MSF) in the conflict affected Walikale territory of North Kivu, Democratic Republic of the Congo (DRC), indicated mortality rates exceeding humanitarian crisis thresholds and a high burden of mortality and morbidity due to malaria. In late 2017, after a period of relative stability, MSF reassessed the health status of the population through a second survey to guide ongoing operations.

**Methods:**

A two-stage cluster survey, selecting villages using probability proportional to size and households using random walk procedures, was conducted. Household members were interviewed on morbidity and mortality, healthcare use, vaccination status, and bednet availability.

**Results:**

The sample included 5711 persons in 794 households. The crude mortality rate (CMR) and under-five mortality rate (U5MR) were 0.98 per 10,000 persons/day (95% confidence interval (CI) 0.78–1.2) and 1.3 per 10,000 persons/day (95% CI): 0.82–2.0), respectively. The most frequently reported causes of death were fever/malaria (31%), diarrhoea (15%) and respiratory infections (8%). In 89% of households at least one person was reported as falling ill in the previous 2 weeks, and 58% sought healthcare. Cost was the main barrier amongst 58% of those who did not seek healthcare. Coverage of measles-containing-vaccine was 62% in under-fives. Sufficient bednet coverage (1 bednet/2 people) was reported from 17% of households.

**Conclusion:**

The second survey illustrates that although mortality is now just below crisis thresholds, the area still experiences excess mortality and has substantial health needs. The study results have supported the further expansion of integrated community case management to improve access to care for malaria, diarrhoea and respiratory infections. Such surveys are important to orient operations to the health needs of the population being served and also highlight the ongoing vulnerability of populations after humanitarian crises.

## Background

In most humanitarian crises, routine and robust health information systems are lacking and, therefore, surveys remain a valuable tool for monitoring the impact of the crisis, assessing health needs of the affected population, and informing and evaluating interventions [1]. These surveys are often the only way of obtaining accurate estimates of important health and health system indicators such as mortality rates, nutritional status and vaccination coverage.

**Fig. 1:**
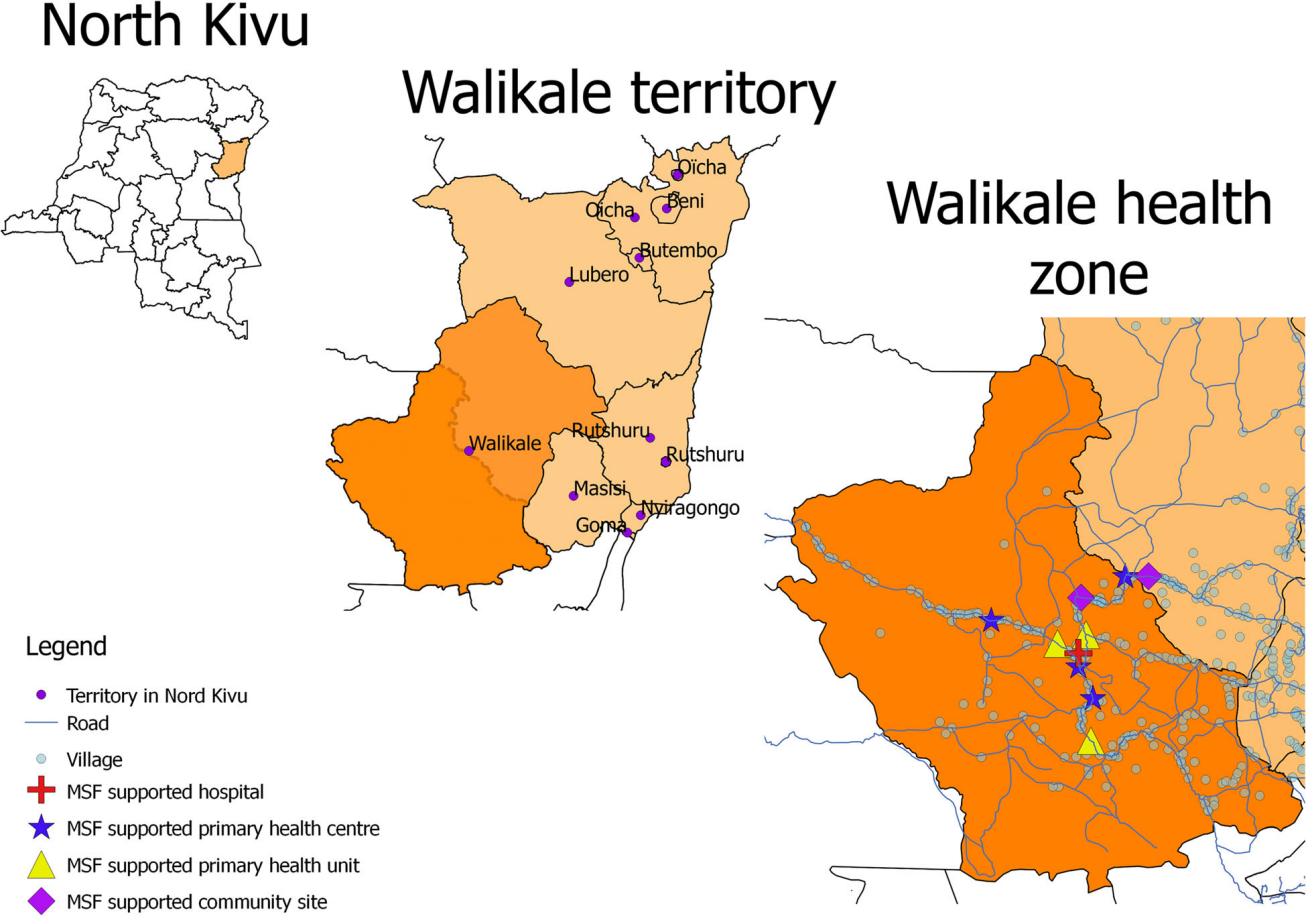
MSF Walikale project area and MSF supported health facilities, North Kivu, DRC

Walikale is a territory in the province of North Kivu, in the east of the Democratic Republic of the Congo (DRC; Fig. 1). The area has experienced decades of political unrest and conflict. Médecins Sans Frontières (MSF), having been previously active in the area between 2003 and 2008, restarted activities in Walikale in 2012 in response to an upsurge of conflict in the area.

In April 2013, a mortality and health survey conducted by MSF in Walikale estimated a crude mortality rate (CMR) of 1.4/10,000 persons/day (95% CI: 1.2—1.7), above the humanitarian crisis threshold level of 1/10,000 persons/day [2, 3] while the under-five mortality rate (U5MR) was 1.9/10,000 persons/day (95% CI: 1.3—2.5), just under the crisis threshold of 2. Malaria accounted for the majority of deaths, while intentional violence accounted for nearly 10% of deaths in those aged over 5 years. The results of this study guided MSF operations in the area: support was extended to more primary health centres with a particular emphasis on child health; four primary health units were supported to treat uncomplicated malaria, diarrhoea and respiratory tract infections; a community led malaria proramme was supported; and support was provided to the paediatric department and referral systems implemented. MSF has also since commenced supporting nutritional, obstetric and laboratory services in the district hospital and a mental health programme. In addition, in 2017 a strategy known as integrated community case management (iCCM) was introduced to bring health care closer to the communities for improved access. It involves the training and support of community health workers (CHWs) to provide treatment beyond formal health facilities [4, 5].

Since 2013, the situation in Walikale has evolved with an abatement in conflict, an increase in the number of previously internally displaced persons (IDPs) returning to the area and an apparent general improvement in the humanitarian situation. In December 2017, after a period of relative stability, we undertook a retrospective health and mortality survey to reassess the health status and access to health care of the population to inform ongoing operations. The primary objective was to estimate the CMR and U5MR in the catchment area of the MSF Walikale project; while secondary objectives included an estimation of morbidity, access to healthcare, and the vaccination coverage and nutritional status of children under five. We present the results of this survey, discuss how they compare to the prior 2013 survey, and describe how the estimates have since been used to inform operations.

## Methodology

### Sampling

A two-stage cluster design was used. Using “ENA for SMART 2011” [6] software it was estimated that a sample size of 800 households was required to calculate a CMR with a precision of 0.3 deaths/10,000 persons/day, based on an expected CMR of 1.0, a precision of 0.3, a design effect (DEFF) of 3, a non-response rate of 10%, and an average household size of 6 persons. The latter three assumptions were based on the prior 2013 study [2]. In order to reach 800 households, a sample of 40 clusters of 20 households each was proposed.

Primary sampling units (PSUs) were villages in the catchment area of the MSF Walikale project which covers the health zone of Walikale and the health area of Lowa in the health zone of Itebero. The sampling frame was developed using data from the last national population census adjusted for estimated annual growth and consisted of 156 villages and with an estimated population of 169,795 persons. Of note, there were no formal IDP camps within the study area at the time of the survey and the majority of displaced persons were sheltered within host communities. After an initial risk assessment, no village was excluded a priori from the sampling frame. Probability proportional to size (PPS) sampling was used to select 40 villages. Five back-up villages were also selected.

Within selected villages, households (i.e. a single structure housing a group of people who regularly sleep and eat together, and who are under the responsibility of one person) were selected using a “random walk” approach. From the geographic centre of the village a direction was chosen at random (by throwing a pen on the ground). The field team walked to the edge of the village along this direction and then chose another direction back towards the village at random. All houses in this second line were enumerated and one selected using a random number table to be the first household to be interviewed. Subsequent households were selected based on physical proximity i.e. the house whose front door was closest. Where two houses were equidistant, the house to the left was selected. Empty households were revisited at least twice during the day of the visit of the interview team. If there was no response by the end of the day it was replaced with the next eligible household.

A head of household was defined as person aged > 15 years present at the time of the survey who had lived in the household regularly during recall period, and could give accurate information on demographic and mortality issues in his/her household. At the household level, all household members during the recall period were considered eligible for inclusion. Households were excluded if a head of household could not be identified or if consent was not obtained. If a household refused to participate it was not replaced. The presence or not of children aged 6—59 months did not influence inclusion overall – only the inclusion of the vaccination questionnaire and the nutritional assessment.

### Data collection

A series of structured interviews were conducted with the head of household. Interviews were conducted in Swahili by trained study teams. The questionnaire was initially piloted with households not included in the final analysis. A mortality questionnaire asked for the age and gender of all those who were household members during the recall period of 324 days (chosen to be comparable with the 2013 study and to correspond to the start of the year to aid recall), and noted any arrivals, departures and deaths. Of note, compared to the 2013 survey, questions on the settlement status of the household were not included. For deaths, the month, respondent-reported cause and place of death was recorded. In the absence of another diagnosis, a fever was categorised as malaria and a fever with a rash as measles. A morbidity questionnaire asked about the last person that was sick in the previous 2 weeks in the household. For this person, the respondent-reported cause of illness was recorded. If healthcare was sought, the timing, place, and reason for choosing this place were recorded. If healthcare was not sought, the reason for this was recorded. A bednet questionnaire asked if there was a bednet in the house, the number of nets and the number of household members who slept under the bednet in the previous night. Of note the questionnaire did not specify if the net was a long lasting insecticide treated net (LLITN). A vaccination questionnaire asked about the vaccination status of children age 0 to 59 months. Three vaccines were considered: Bacillus Calmette–Guérin (BCG) vaccine, oral polio vaccine (OPV) and measles containing vaccine (MCV). The number of OPV doses was not asked, only if at least one dose had been received. Vaccination status was extracted from the vaccination card if available, or otherwise based on recall. The site of injection and age was used by the interview team to assist recall. A rapid nutritional assessment was conducted on children aged six to 59 months who were present at the time of the interview. The assessment consisted of a check for the presence of pitting oedema and measuring the mid upper arm circumference (MUAC) with MUAC strips. Interviews were conducted between 4 and 9 December 2017.

### Data analysis

Excel was used to collate data from the paper questionnaires into an electronic database. STATA 14 (StataCorp, College Station, TX, USA) was used for data analysis using the svyset command to take account of any cluster effect. The DEFF and 95% CIs were calculated. Data checks and cleaning was performed prior to analysis. Mortality rates were estimated as the number of deaths per 10,000 population per day. The mid-period population size (household members at end of study period + 0.5(deaths)–0.5(births)) was used as the denominator as exact dates of arrival and departure were not collected. Vaccination coverage estimates were restricted to the age groups eligible for the specific vaccine as per the national schedule of the DRC [7].

### Ethical considerations

The study utilised a protocol preapproved by the MSF Ethics Review Board (ERB) [8]. The MSF Medical Director determined that this project fulfilled the preapproval criteria [9]. Permission to undertake the survey was obtained from the local health authority and the Ministry of Health (MoH) of North Kivu. The heads of selected villages were informed of the study and their approval was sought before proceeding. The heads of selected households were provided with oral and written information about the study, and oral consent was obtained.

## Results

A total of 794 households were included. Two of the originally selected villages were substituted by back-up villages due to a combination of logistical and security reasons. Seven households from different villages refused to participate. In one village an extra household was included in error but retained for the analysis. In the included households, the number of household members during the recall period was 5711 – 5031 were present at the start of the recall period and the number of current household members was 5288. There were 680 arrivals – 233 births and 447 other arrivals; and 423 departures – 164 deaths and 259 other departures. The average household size was 6.7 persons.

**Fig. 2:**
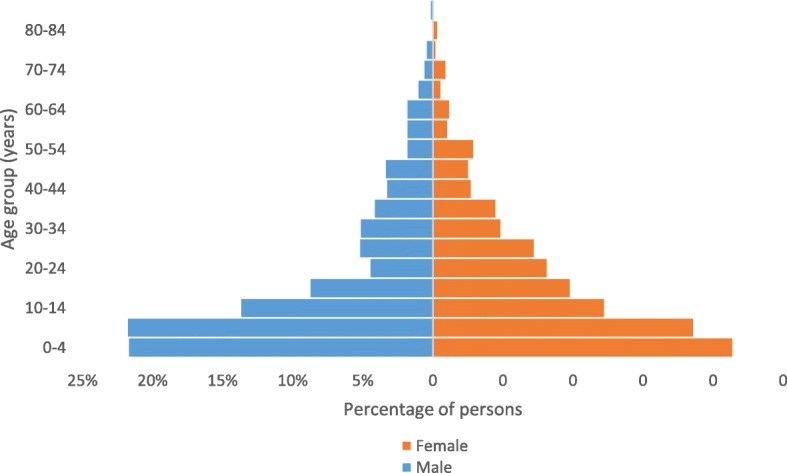
Age distribution of current household population by five year age groups and by gender; mortality and health survey, MSF Walikale project, 2017

The median age of the current population was 12 years (range 0–90 years). Females accounted for 52.6% (95% CI 51.3—53.9) of the population, while children under-five accounted for 21.6% (95% CI 20.4—22.8). The male to female ratio was 0.9 overall, with the lowest ratio of 0.5 seen in the 20 to 24 years age group (Fig. 2). The proportion of pregnant women in the current population was 3.2% (95% CI 2.6—3.8), and amongst women aged 15 to 49 years it was 15.1% (95% CI 12.6—17.9%).

### Mortality

During the recall period, a total of 164 deaths were reported. Of these, 26% (*n* = 43; 95% CI 18.5—35.8) were children under-five, and 59.8% (*n* = 98; 95% CI 51.9—67.1) were males.

The CMR and U5MR were 0.98 deaths/10,000 persons/day (95% CI 0.78—1.23; DEFF 2.1) and 1.29/10,000 persons/day (95% CI 0.82—2.00; DEFF 2.1), respectively. Overall, the most frequent respondent-reported causes of death were fever/malaria (31.1%; 95% CI 22.7—40.9), followed by diarrhoea (14.6%; 95% CI 8.3—24.6), and respiratory infections (7.9%; 95% CI 4.7—13.1) (Table 1).

**Table 1:** Respondent-reported causes of death and cause-specific mortality rates, MSF Walikale project, 2017

Reported cause of death	N	%	95% CI	Cause-specific mortality per 10,000 persons/day	95% CI
Fever/malaria	51	31.1	22.7—40.9	0.30	0.19—0.48
Diarrhoea	24	14.6	8.3—24.6	0.14	0.08—0.26
Respiratory infection	13	7.9	4.7—13.1	0.08	0.05—0.13
Trauma/accident	11	6.7	3.5—12.4	0.07	0.03—0.13
Measles	11	6.7	3.6—12.1	0.07	0.04—0.12
Pregnancy related	4	2.4	0.9—6.2	0.02	0.01—0.06
Perinatal/neonatal (infant)	3	1.8	0.6—5.5	0.02	0.01—0.06
Violence	1	0.6	0.1—4.5	0.01	0.00—.05
Don’t know	7	4.3	2.1—8.5		
Other	39	23.8	17.0—32.2		

Amongst children under-five, fever/malaria accounted for 51.2% (95% CI 35.8—66.3) of deaths, while diarrhoea accounted for 14.0% (95% CI 6.8—26.6) (Table 2). In those aged over five, fever/malaria accounted for 24.0% (95% CI 14.9—36.2) of deaths and diarrhoea accounted for 14.9% (95% CI 7.1—28.4).

**Table 2:** Respondent-reported causes of death by age group, MSF Walikale project, 2017

Reported cause of death	< 5 years			> = 5 years		
	n	%	95% CI	n	%	95% CI
Fever/malaria	22	51.2	35.8—66.3	29	24.0	14.9—36.2
Diarrhoea	6	14.0	6.8—26.6	18	14.9	7.1—28.4
Respiratory infection	3	7.0	2.1—20.4	10	8.3	4.7—14.1
Trauma/accident	1	2.3	0.3—15.9	10	8.3	4.3—15.4
Measles	0	–		11	9.1	5.0—15.9
Pregnancy related	n/a			4	3.3	1.3—8.2
Neonatal/perinatal	3	7.0	2.6—17.3	n/a		
Violence	0	–	–	1	0.8	0.1—6.2
Don’t know	4	9.3	3.5—22.5	3	2.5	0.8—7.5
Other	4	9.3	2.7—27.3	35	28.9	21.2—38.1
Total	43			121		

The proportion of “other” causes of death increased with age. In children under-five “other” causes of death included anaemia, epilepsy and jaundice. In those over five “other” causes included: hernia (*n* = 4), asthma (*n* = 3), blood loss (*n* = 3), epilepsy (*n* = 2), malnutrition (*n* = 2), cancer, diabetes, hepatitis, old age, hypertension, gastritis, meningitis, skin infection, vomiting, and post-surgery (all *n* = 1). Of note, in this part of DRC the term “hernia” is used to describe a range of symptoms or diseases and may not mean a true hernia.

Pregnancy related deaths accounted for 6.1% (95% CI 2.4—14.7) of all female deaths. Amongst females of reproductive age (15–49 years) they were the most commonly reported cause of death (22%; 95% CI 8.6—46.5).

### Morbidity

In the 2 weeks preceding the interview, 709 households (89%; 95% CI 86—92; DEFF 1.4) reported that at least one household member had been ill. Fever/malaria was the most frequently reported type of illness overall, accounting for over half of illnesses (57%; 95% CI 52—62), followed by respiratory infections (12%; 95% CI 9.0—16) and diarrhoea (11%; 95% CI 9—13). Fever/malaria and diarrhoea were also the most frequent illnesses amongst under-fives and over-fives (Table 3). There was a higher proportion of “other” illnesses in those aged over five – “general pain” (*n* = 24) and abdominal problems (*n* = 23) accounted for the majority of these. In children under-five, anaemia, malnutrition and abdominal problems accounted for all “other” causes.

**Table 3:** Reported causes of illness for the last person ill in the previous 2 weeks in the household overall and by age; mortality and health survey, MSF Walikale project, 2017

	Total			Households where last person ill was < 5			Households where last person ill was > =5		
	n	%	95% CI	n	%	95% CI	n	95% CI	
Fever/malaria	402	56.7	51.7–61.5	152	58.5	50.8–65.8	250	55.8	49.5–61.7
Respiratory infection	85	12.0	9.0–15.9	45	17.3	12.1–24.1	40	8.9	5.8–13.4
Diarrhoea	76	10.7	8.5–13.4	41	15.8	11.5–21.3	35	7.8	5.2–11.5
Other infectious	34	4.8	3.0–7.5	10	3.8	2.0–7.3	24	5.3	3.0–9.3
Pregnancy related	10	1.4	0.8–2.4				10	2.2	1.3–3.8
Trauma/accident	7	1.0	0.5–2.0	1	0.4	0.0–2.9	6	1.3	0.6–2.9
Measles	2	0.3	0.1–1.2	1	0.4	0.0–2.9	1	0.2	0.0–1.7
Other	93	13.1	10.6–16.1	10	3.8	2.0–7.3	83	18.4	14.6–23.1

### Access to healthcare

Amongst households reporting an illness in the previous 2 weeks, healthcare was sought in 58% (95% CI 50—64; DEFF 3.5) of cases. Table 4 shows the proportion seeking healthcare by cause of illness, age group and gender. The difference in the proportion seeking healthcare was not significantly different for any of these factors.

**Table 4:** Number and proportion of cases seeking healthcare by cause of illness; mortality and health survey, MSF Walikale project, 2017

	n/N	% (95% CI)	*P* value
Cause of illness			*p* = 0.652
Fever/ malaria	240/402	59.7 (51.1–67.7)	
Respiratory infection	46/85	54.1 (39.7–67.9)	
Diarrhoea	34/76	44.7 (34.7–55.2)	
Other infectious	21/34	59.1 (0.47–69.8)	
Pregnancy related	6/10	60.0 (51.1–67.7)	
Trauma/ accident	6/7	85.7 (39.6–98.2)	
Measles	0/2	–	
Other	55/93	61.8 (41.4–78.7)	
Age-group			*p* = 0.382
0 to 11 months	30/47	63.8 (48.0–77.1)	
1 to 4 years	135/213	63.4 (53.9–71.9)	
5 to 14 years	83/62	51.2 (40.7–61.7)	
15 to 60 years	140/249	56. 2 (48.0–64.1)	
60+ years	20/38	52.6 (35.3–69.3)	
Gender			*p* = 0.383
Male	180/302	59.6 (52.2–66.6)	
Female	228/407	56.02 (48.0–63.7)	

Amongst those who sought healthcare, it was on the day of illness onset in 41% (95% CI 36–47), or within two to 7 days in 53% (95% CI 47—59). Table 5 shows the time to seeking care for by cause of illness, age-group and gender

**Table 5:** Time to seeking care for the last ill member of the household in the previous 2 weeks by cause of illness, age-group and gender; mortality and health survey, MSF Walikale project, 2017

	Same day		2 to 7 days		Over 8 days		
	n	1% (95% CI)	n	1% (95% CI)	n	1% (95% CI)	
Cause of illness							< 0.001
Fever/ malaria	113	47.3 (40.6–54.1)	122	51.0 (44.7–57.3)	4	1.7 (0.6–4.3)	
Respiratory infection	17	37.0 (21.7–55.3)	28	60.9 (44.2–75.3)	1	2.2 (0.3–13.8)	
Diarrhoea	14	41.2 (26.7–57.4)	20	58.8 (42.6–73.3)			
Pregnancy related	3	50.0 (15.8–84.2)	2	33.3 (7.8–74.7)	1	16.7 (2.1–65.4)	
Trauma/ accident	3	50.0 (15.8–84.2)	3	50.0 (15.8–84.2)			
Other infection	5	23.8 (9.8–47.3)	12	57.1 (30.9–79.9)	4	19.0 (6.0–46.6)	
Other	12	23.5 (12.2–40.5)	28	54.9 (36.1–72.4)	11	21.6 (11.1–37.6)	
Age-group							0.075
Infant	16	53.3 (38.3–67.8)	14	46.7 (32.2 (61.7)		0.0	
1 to 4 years	55	40.7 (32.8–49.2)	76	56.3 (47.3–64.9)	4	0.3 (0.9–9.2)	
5 to 14 years	42	51.2 (38.0–43.7)	38	46.3 (33.3–59.8)	2	2.4 (0.6–10.0)	
15 to 60 years	49	35.8 (28.6–43.7)	76	55.5 (47.0–63.6)	12	8.8 (4.6–15.9)	
60+ years	5	26.3 (10.7 (51.5)	11	57.9 (36.5–76.7)	3	15.8 (5.4–38.1)	
Gender							0.479
Male	71	39.4 (31.5–47.9)	97	53.9 (44.9–62.6)	12	6.7 (3.5–12.4)	
Female	96	43.0 (36.4–49.9)	118	52.9 (46.4–59.4)	9	4.0 (2.1–7.8)	
	167		215		21	100%	

In cases where healthcare was not sought, consultation costs was cited as the reason by 58% (95% CI 50—66) and seeking drugs at the market or pharmacy was cited by 49% (95% CI 40—58).

Amongst those who died, 60% (95% CI 49—70) died in a health facility and 39% (95% CI 29—50) died in the community. Included in community deaths are 6.7% (95% CI 3.7—12) of deaths which occurred on-route to a health facility. A traditional or religious site was the place of death for 1.2% (95% CI 0.3—4.5) of deaths. Table 6 shows the place of death by reported cause of death.

**Table 6:** Proportion of deaths occurring in a healthcare facility by cause of death; mortality and health survey, MSF Walikale project, 2017

	Health facility		Community		Traditional or religious place	
	n	% (95% CI)	n	% (95% CI)	n	% (95% CI)
Diarrhoea	15	62.5 (42.0–79.3)	9	37.5 (20.7–58.0)		
Respiratory infection	6	46.2 (23.0–71.1)	7	53.8 (28.9–77.0)		
Fever/ malaria	40	78.4 (63.4–88.4)	10	19.6 (9.4–36.5)	1	2.0 (0.3–11.5)
Pregnancy related	1	25.0 (3.0–78.2)	3	75.0 (21.8–97.0)		
Neonatal /perinatal	3	100.0		0.0		
Trauma/ accident	4	36.4 (10.9–72.7)	7	63.6 (27.3–89.1)		
Violence	0	0.0	1	100.0		
Measles	5	45.5 (21.3–72.0)	6	54.5 (28.0–78.7)		
Don’t know	5	71.4 (36.9–91.5)	1	14.3 (2.5–52.1)	1	14.3 (1.7–61.8)
Other	19	48.7 (33.3–64.4)	20	51.3 (35.6–66.7)		

Table 7 shows the proportion of deaths in a health facility by age. Amongst infants, 90.9% (95% CI 57.8–98.6) of deaths occurred in a health facility compared to 34.3% (95% CI 18.6–54.3%) of deaths in those aged over 60.

**Table 7:** Proportion of deaths occurring in a health facility by age group; mortality and health survey, MSF Walikale project, 2017

Age group	n	% (95% CI)
0 to 11 months	10/11	90.9 (57.8–98.6)
1 to 4 years	26/32	81.3 (62.1–92.0)
5 to 14 years	18/27	66.7 (44.6–83.3)
15 to 60 years	32/59	54.2 (39.2–68.5)
60+ years	12/65	34.3 (18.6–54.3)

### Vaccination coverage

Information on vaccination status was recorded for 1147 children aged 0 to 59 months. A vaccination card was available for 11% of children. Crude vaccination coverage (i.e. by vaccination card or recall) was 83% (95% CI 78—87%) for BCG amongst children age 0 to 59 months, 94% (95% CI 91—96%) for at least one dose of OPV amongst children aged 2 to 59 months, and 67% (95% CI 61—73%) for MCV amongst children aged 9 to 59 months (Table 8).

**Table 8:** Vaccination coverage by antigen and source of information, MSF Walikale project, 2017

Vaccine (age included in analysis)	By history		By card		Total		
	%	95% CI	%	95% CI	%	95% CI	DEFF^b^
BCG (0–59 months)	73.3	67.7—78.3	9.4	6.0–14.4	82.7	77.6—86.9	4.2
OPV^a^ (2–59 months)	84.6	79.7—88.6	8.9	5.6–13.8	93.5	90.7—95.6	2.6
MCV (9–59 months)	61.9	55.6—67.8	5.5	3.1–9.7	67.3	60.6—73.4	4.4

^a^At least one dose

^b^DEFF – design effect

### Bednet coverage

At least one bednet was reportedly available in 73.9% (95% CI 66.6—80.1; DEFF 4.6) of households. The average number of reported bednets per household was 2.0 (95% CI 1.8—2.2; SD 1.3). The proportion of houses with at least one bednet per two people (i.e. sufficient coverage) was 17% (95% CI 14—22). Amongst those sleeping in the house the previous night, 49% (95% CI 42—56) reportedly slept under a bednet. Amongst the key risk groups, 69% (95% CI 61—75) of infants, 52% (95% CI 44—60) of children under-five, and 57% (95% CI 46—68) of pregnant women reportedly slept under a bednet.

### Nutritional status

The result of the nutritional assessment was available for 934 children aged 6 to 59 months. The overall prevalence of global and severe acute malnutrition (GAM, SAM) was 2.5% (95% CI 1.6—3.9; DEFF 1.2) and 0.4% (95% CI 0.2—1.1; DEFF 0.9) respectively, and 3.8% (95% CI 1.9—7.5; DEFF 1.1) and 0.8% (95% CI 0.2—3.5; DEFF 1.0) respectively in children less than 2 years of age (Table 9).

**Table 9:** Acute malnutrition amongst children 6 to 59 months and under 2 years; MSF Walikale project, 2017

Level of malnutrition	All			Children under 2 years		
	n	%	95% CI	n	%	95% CI
Global acute malnutrition (MUAC < 125 mm or oedema)	23	2.5	1.6—3.9	9	3.8	1.9—7.5
Moderate acute malnutrition (MUAC 115 mm–124 mm)	19	2	1.2—3.4	7	3	1.3—6.6
Severe acute malnutrition (MUAC < 115 mm or oedema)	4	0.4	0.2—1.1	2	0.8	0.2—3.5

**Table 10:** Comparison of data available from the 2013 and 2017 mortality and morbidity surveys, MSF Walikale project

	2017	2013
Demographics		
Median age	12	15
Male to female ratio	0.9	1.0
Mean household size (95% CI)	6.7	6.4
Mortality		
Mortality rates (Deaths per 10,000 persons/day (95% CI))		
CMR	0.98 (0.78–1.2)	1.4 (1.2–1.7)
U5MR	1.3 (0.8–2.00)	1.9 (1.3–2.5)
Reported cause of death - overall (% (95% CI))		
Fever/malaria	31.1 (22.7–40.9)	34.1 (25.4--42.9)
Diarrhoea	14.6 (8.3–24.6)	16.2 (8.8–23.6)
Respiratory infection	7.9 (4.7–13.1)	7.5 (3.7–11.3)
Trauma/accident	6.7 (3.5–12.4)	5.8 (1.2–10.3)
Measles	6.7 (3.6–12.1)	2.9 (1.1–6.3)
Pregnancy related	2.4 (0.9–6.2)	6.4 (2.4–10.3)
Perinatal/neonatal (infant)	1.8 (0.6–5.5)	nr
Violence	0.6 (0.1–4.5)	7.5 (3.9–11.1)
Hernia^a^	nr	4.6 (2.2–8.6)
Don’t know/missing	4.3 (2.1–8.5)	0.6 (0.6–1.8)
Other	23.8 (17.0–32.2)	14.5 (9.8–20.3)
Reported cause of death - < 5 years (% (95% CI))		
Fever/malaria	51.2 (35.8–66.3)	56.8 (38.4–75.3)
Diarrhoea	14 (6.8–26.6)	18.2 (5.9–30.5)
Respiratory infection	7 (2.1–20.4)	4.5 (−2.4–11.5)
Trauma/accident	2.3 (0.3–15.9)	2.3 (−2.5–7.0)
Measles	–	11.4 (4.3–23.4)
Neonatal/perinatal	7 (2.6–17.3)	nr
Violence	–	2.3 (−2.5–7.0)
Don’t know/missing	9.3 (3.5–22.5)	4.5 (0.8–14.2)
Other	9.3 (2.7–27.3)	–
Reported cause of death - > = 5 years (% (95% CI))		
Fever/malaria	24 (14.9–36.2)	26.4 (16.0–36.7)
Diarrhoea	14.9 (7.1–28.4)	15.5 (8.4–22.7)
Respiratory infection	8.3 (4.7–14.1)	8.5 (4.7–12.4)
Trauma/accident	8.3 (4.3–15.4)	7.0 (1.0 (13.0)
Measles	9.1 (5.0–15.9)	
Pregnancy related	3.3 (1.3–8.2)	8.5 (3.0 (14.0)
Violence	0.8 (0.1–6.2)	9.3 (4.6 (14.0)
Hernia^a^	nr	6.2 (2.9–11.4)
Don’t know/ missing	2.5 (0.8–7.5)	0.8 (20.8–2.4)
Other	28.9 (21.2–38.1)	17.8 (11.9–25.2
Morbidity		
At least one ill person in household (% (95% CI))	89.3(86.3–91.7)	89.0 (85.4–92.8)
% under five	36.7 (32.4–41.1)	40.3 (40.3–44.4)
Type of illness - Overall (% (95% CI))		
Fever/malaria	56.7 (51.7–61.5)	56.4 (52.2–60.5)
Respiratory infection	12.0 (9.0–15.9)	4.0 (2.4–5.5)
Diarrhoea	10.7 (8.5–13.4)	17.9 (14.7–21.1)
Other infectious	4.8 (3.0–7.5)	Nr
Pregnancy related	1.4 (0.8–2.4)	7.6 (5.2–10.0)
Trauma/accident	1 (0.5–2.0)	0.9 (0.1–1.6)
Measles	0.3 (0.1–1.2)	–
Other	13.1 (10.6–16.1)	13.1 (9.0–17.2)
Type of illness - < 5 years (% (95% CI)		
Fever/malaria	58.5 (50.8–65.8)	65.0 (57.6–72.4)
Respiratory infection	17.3 (12.1–24.1)	3.8 (0.8–6.8)
Diarrhoea	15.8 (11.5–21.3)	26.9 (20.5–33.4
Other infectious	3.8 (2.0–7.3)	
Trauma/accident	0.4 (0.0–2.9)	
Measles	0.4 (0.0–2.9)	
Other	3.8 (2.0–7.3)	4.3 (1.4–7.2)
Type of illness - > =5 years (% (95% CI))		
Fever/malaria	55.8 (49.5–61.7)	50.6 (44.4–56.7)
Respiratory infection	8.9 (5.8–13.4)	4.0 (2.4–5.7)
Diarrhoea	7.8 (5.2–11.5)	11.8 (8.1–15.6)
Other infectious	5.3 (3.0–9.3)	
Pregnancy related	2.2 (1.3–3.8)	12.7 (8.4–17.1)
Childbirth related	nr	0.3(−0.3–0.9)
Trauma/accident	1.3 (0.6–2.9)	1.4 (0.2–2.7)
Measles	0.2 (0.0–1.7)	
Other	18.4 (14.6–23.1)	19.1 (13.3–24.9)
Healthcare Access		
Sought healthcare amongst person ill	57.5 (50.4–64.3)	63.8 (57.1–70.5)
Reasons for not seeking care (% (95% CI))		
Consultation costs	58.3 (50.1–66.1)	63.8 (52.2–75.4)
Sought drugs at market or pharmacy	48.7 (39.9–57.5)	40.0 (52.2–75.4)
Too far	12.0 (7.8–18.1)	
Transport costs	9.0 (5.1–15.4)	
Not considered sick enough	6.0 (3.2–10.9)	7.1 (2.4–11.9)
Lack of confidence	4.3 (1.9–9.8)	
Used traditional medicine	2.7 (1.4–5.0)	10.0 (5.7–14.3)
Lack of time	1.3 (0.5–3.5)	
Lack of medicines in health facilities	1.33 (0.4–4.4)	
Other priorities	0.7 (0.2–2.8)	
Refused care	0.3 (0.0–2.5)	
Security		0.5 (0.5–1.4)
Bednet Availability		
Household owns at least 1 bednet (% (95% CI))	73.9 (66.6—80.1)	56.5 (49.7–63.3)
Average number of bednets per household	2.0 (1.8–2.2)	1.6

*nr* not reported

^a^“hernia” is used to describe a range of symptoms or diseases and may not mean a true hernia

## Discussion

This is the second mortality and morbidity study undertaken by MSF in the Walikale project area. The first in 2013 was undertaken during a time of intensified conflict. These sequential studies allow for a valid comparison of health indicators over time and a more reliable identification of changing health needs, given the changed socio-political context. In addition to its value for operational planning, this study also provides us with a better understanding of the prolonged impact of conflict on health. Such mortality studies are most commonly undertaken at the onset of a crisis, where they have an important role in documenting the impact of the crisis and also in advocacy for humanitarian assistance or conflict resolution [10, 11]. These surveys are not as common in the post humanitarian emergency phase. This follow-on study highlights the continued need for accurate health status estimates, not only to inform ongoing operations but also to highlight the continued vulnerability of populations in the post conflict era.

The CMR has significantly decreased since 2013. However it is still resting just below the crisis threshold level with the upper confidence limit extending beyond it. Similarly, the U5MR has decreased since 2013, although not significantly, and its upper confidence limit just reaches the emergency threshold level. These reductions in mortality suggest some improvement in the humanitarian situation in Walikale. However, the CMR and U5MR still exceed those of sub-Saharan Africa (0.41 and 1.07 respectively) and the DRC [12–15], and coupled with the persistent high morbidity, suggest that conditions remain precarious in the region.

Similarly to the 2013 survey, and national mortality patterns for the DRC, the most common causes of death were fever/malaria, followed by diarrhoea and respiratory infections [16]. The high proportion of fever/malaria deaths seen in this study may be due to the lack of specificity of this category as a proxy indicator for malaria. Fever is a symptom of many infections. Therefore, the burden of malaria is likely to be overestimated. Nearly 80% of deaths from fever/malaria did occur in a healthcare facility, and 60% with fever/malaria illness attended a health facility. However we do not know if the diagnosis was confirmed by a diagnostic test at the facility.

There was a particularly notable decrease, although not reaching statistical significance, in mortality due to intentional violence (0.6% (95% CI 0.1–4.5) vs 10% (95% CI 3.9—11.1) of deaths). With the dispansion of some of the armed groups in the area and the remission in conflict the decrease in violent deaths is not unexpected.

Pregnancy related deaths also appear to have decreased (2.4% (95% CI 0.9—6.2) vs 6.4% (95% CI 2.4—10.3) of all deaths). This decrease is consistent with a national decreasing trend in maternal mortality [17]. Similarly, pregnancy related morbidity has decreased, now responsible for 1.4% (95% CI 0.8–2.4) of all illnesses compared to 7.6% (95% CI 5.2–10.0) in 2013. While these improvements are encouraging, more than 1 in 5 deaths in women of reproductive age in the study was pregnancy related. Only half of those reporting pregnancy related illness sought care, and amongst those it was on the day of onset for only half. In addition, 3 of the 4 pregnancy related deaths occurred in the community. While we do not have any further details on these deaths or illnesses (e.g. exact cause or gestation) it does highlight unmet needs in maternal and reproductive healthcare in the region. In early 2019, MSF supported the opening of a new maternity waiting home close to the hospital, where women with high risk pregnancies can stay while awaiting labour and thus reduce perinatal deaths in the community. Also, the adoption by the DRC of the Protocol to the African Charter on Human and People’s Rights on the Rights of Women in Africa, or the Maputo Protocol, in 2018 now enables MSF to provide termination of pregnancy services in specific circumstances such as when the mothers health is endangered by the pregnancy [18].

Morbidity in the previous 2 weeks remains high and unchanged since 2013, and is also largely attributable to malaria, respiratory infections and diarrhoea A slightly lower, but not statistically significant, proportion of households with an ill person in the previous 2 weeks sought healthcare than seen in 2013 (57.5% (95% CI 50.4—64.3) vs 63.8% (95% CI 57.1—70.5)). Cost was the main barrier for not seeking healthcare. The 2017 survey did not ask if those who sought healthcare had to pay, but in 2013 66.4% did have to pay. The survey also did not distinguish between MSF supported facilities and others. While MSF facilities are free to access, it is important to note that at the time of the survey MSF supported only 4 of 16 PHCs in the area, and other PHCs require out of pocket payments. Indirect costs such as for transport may also influence health seeking behaviours.

Similar to 2013, purchasing medications in the market or pharmacy was a commonly cited reason for not seeking healthcare. While self-care, including self-medication, for less serious conditions are important components of healthcare, given the lack of regulation on the sales of medicines in the DRC, the quality and suitability of any medicines cannot be guaranteed [19, 20]. Furthermore, it also poses a risk for the development of anti-malarial or antibiotic resistance at a population level. There are ongoing efforts to improve the regulation of medications in the DRC [21, 22]. Improved access to care will also reduce reliance on self-medication.

There was an increase in the proportion seeking healthcare on the day of illness since 2013 (41% vs 26%) which may reflect better access or better awareness of the need for prompt treatment for certain conditions amongst the population. The proportion seeking care, and the proportion seeking care on the day of illness, was highest for fever/malaria. This may reflect greater awareness of the need for prompt treatment for malaria as a result of the work on malaria by MSF and the MoH in the area since 2012. The lower proportion seeking care, and seeking care on the day of illness, for diarrhoea and respiratory infections does not necessarily reflect poor access or lack of knowledge. In many circumstances these are self-limiting illness and self-care is appropriate. However, awareness of red flags and access to care if these are present is important. Given that these are still responsible for a high proportion of deaths, it suggests that such awareness and access is lacking. 

Time to seeking care was longer for those with other infections and “other” illnesses with 19 and 22% respectively waiting more than 1 week to seek care. This may be because these other types of illness may not present as acutely and it may take longer for symptoms to warrant seeking healthcare. It may also be because there is a perception that care for such illnesses is not as readily available as for the major common infectious diseases. This is in fact true as a number of health facilities only provide care for the three common infections. There was also a trend in waiting longer to seek care with increasing age. This probably relates to the greater proportion of these “other” illness in older age groups.

Place of death is a proxy for healthcare access amongst people who died, although it does not take into account people who attended healthcare but were discharged or the time between illness onset and presentation to a health facility. Though differences in place of death are not statistically significant, 78% of deaths from fever/malaria occurred in hospital compared to 63% of diarrhoeal deaths and 46% of deaths from respiratory infections. As discussed above this may reflect greater knowledge on malaria in the community. The proportion dying in a health facility decreased with age. This may be due to the type of illness resulting in the death and a perception that care is not needed or available. It may also be due to a greater expectation and acceptance of death with increasing age. It is not possible to comment on the potential quality of care received in a health facility based on the proportion of deaths occurring in a facility. This would require information on number of admissions, the severity of illness at presentation and on overall admission rates.

Since 2017, MSF has supported an iCCM programme in Walikale. iCCM aims to improve access to timely and effective treatment for the three key illnesses of malaria, diarrhoea and respiratory infections, by extending care beyond fixed health facilities [4, 5]. MSF has been working with the MoH and the Bureau Central de Zone de Santé to train community health workers in North Kivu to identify and treat these key illnesses in the community. This strategy will also decrease indirect costs of seeking healthcare and will mitigate against the financial barriers to care. However, this study also highlights that in addition to programs tackling the major infectious diseases, there are also needs around non-communicable diseases, particularly amongst older age groups.

While there was a statistically significant increase in the proportion of households which reported owning at least one bednet (73.9% (95% CI 66.6—80.1) vs 57% (95% CI 49.7—63.3)), and the number of bednets per household has increased from 1.6 in 2013 to 2.0, coverage remains suboptimal with less than one in five households having enough bednets for all household members and less than half of persons sleeping under a bednet the previous night. These estimates and the evidence provided by this same study of the high burden of fever/malaria, highlight the importance to continue activities on bednet distribution. MSF distributes bednets to women attending antenatal clinics, children attending ambulatory or inpatient therapeutic feeding centres, children under-five attending paediatric services, and at blood donation centres. Notably the proportion of those age 5 to 14 years sleeping under a bednet was considerably lower than other age groups. Fever/malaria accounted for 48% of deaths in this age group. The reason for the low bednet use in this age group is not explored in this study. It may be due to preferential use of household bednets in younger age-groups or poor compliance amongst this age group even if a bednet is available to them. A specific knowledge, attitudes and practice survey on malaria and malaria prevention may give a better insight on bednet use in order to target future efforts.

The suboptimal vaccination coverage of MCV, coupled with the absence of a second dose of MCV as part of national schedule as recommended, renders the population highly vulnerable to measles outbreaks [23]. In August 2018, several clusters of measles emerged in Walikale. The availability of these recent and precise estimates on vaccine coverage facilitated the risk assessment by MSF which determined the risk of a large and widespread measles outbreak as high, and supported a supplementary vaccination campaign which reached 31,000 children. The difference in coverage between BCG (83%), recommended at birth, and MCV1, recommended at 9 months, indicates a high dropout rate. Dropout over longer periods is generally considered an indicator of programme effectiveness, while dropout over short intervals more related to caregivers utilization or access to services. While further studies are required to understand reasons for non-vaccination specific to this area, the routine immunization systems evidently requires considerable strengthening. The nutritional estimates found by this survey were below the crisis thresholds [24] even though the survey was undertaken at the end of the lean season and before the first harvest when food stocks would typically be at their lowest [25]. In the months following this study there was an increase in admissions to MSF supported nutritional programmes and nutritional activities were upscaled in response. This increase was attributed to the cessation of a malnutrition programme operated by another actor in a number of villages in the area, rather than a true deterioration in nutritional status. The classification of the food security situation in North Kivu province below crisis levels in the first months of 2018 [26] and more importantly the availability of the recent and more granular estimates (i.e. health zone levels) for Walikale provided by this study, provided further reassurance to MSF. It is important to note that although the study did not highlight acute malnutrition as an issue, previous studies identified a high prevalence of chronic malnutrition in the province. In Walikale the relative stability in recent years may have improved food security. However, the food security situation across the majority of the territory was classified by the World Food Programme as “stressed” during 2017 [27]. Therefore nutritional monitoring and nutritional programmes are still important in the area.

A final point of interest is that, similarly to the 2013 study, the male to female ratio is lowest amongst those aged 20 to 29 years. The exact reasons for this are not known. In the current survey, migration amongst men for work may be responsible. Increased displacement of men due to conflict may have also been a contributing factor in the earlier survey. Given the recent stability this is not thought to be factor at present. However, the earlier conflict may still have an impact on the population structure. Despite this, a higher proportion of deaths occurred amongst males. There was a higher proportion of trauma related deaths and morbidity amongst males than females which may reflect the type of work they undertake. However there was also a higher proportion of “other” causes of death which may reflect less health seeking behaviour amongst male adults than females.

### Limitations

The study is limited by a number of factors. First, given the recent population movements in the area, the sampling frame may not have adequately reflected the current study population. Recently expanded villages, which may have a worse health status and worse access to healthcare or preventive interventions, may have been underrepresented. The replacement of two clusters due to logistical and security reasons may have introduced selection bias. These replaced clusters were extremely remote and may have had a worse health status. Secondly, the random walk method used to select households poses a risk of selection bias by field staff. Despite its limitations [10, 28], this method is considered acceptable by many in challenging situations such as those in Walikale where resource, logistical and security constraints can limit the use of more robust sampling methodologies [29, 30].

Another limitation was the reliance on verbal reporting by the household head. 

Reporting bias may also have been present due to lack of knowledge or a desire to give a socially acceptable answer. Reported causes of death or morbidity were not verified through other sources. The limitations of verbal reporting and the use of certain symptoms as proxies have already been discussed in relation to fever as a proxy for malaria. This limitation is also evident in relation to measles, which was reported as the cause of 7% of deaths, almost exclusively in adults. These deaths are likely to have been misreported as there was no evidence from the study of measles morbidity or mortality amongst younger children suggesting that there was not a high level of measles circulation in the area during the recall period. The verbal reporting and the classifications offered also resulted in a high proportion of “other” causes of death and illness, some of which may have been misclassified e.g. deaths due to blood loss may been due to violence or accidental trauma. The long recall period for mortality may have contributed to recall bias. However this duration was chosen to be comparable with the previous study. Recall bias may have particularly impacted on vaccination coverage estimates given the low proportion of children for whom a vaccination card was available. Although site of injection and age was used to aid recall, the fact that children up to 5 years were included and the presence of multiple eligible children per household, recall was likely suboptimal.

It is also important to note the study was not powered to detect statistically significant differences with the 2013 study for its secondary objectives, and changes since 2017 may not have reached statistical significance despite real differences existing.

## Conclusion

This second mortality and health survey in the Walikale MSF project area allowed us to show that although conflict has stabilised and mortality indicators suggest improvements, the area still suffers from excess mortality, has substantial health needs and a health system still requiring considerable strengthening. Conflict is recognised as having a prolonged impact on health, often with a disproportionate impact on women and children [31–33]. Its impact is largely indirect due to poor living conditions and weak or absent healthcare systems. This is evident from our two surveys. During both periods preventable and treatable infectious diseases predominated. These results justify a continued focus on these key diseases by MSF in Walikale.

This study also demonstrates the value of such field surveys in providing accurate and localised epidemiological indicators to inform humanitarian programme planning in settings still lacking other health information systems. Mortality and health surveys should be used to identify, as precisely as possible, the health needs of the population being served both during and after humanitarian crises.

## Data Availability

The datasets used and/or analysed during the current study are available from the corresponding author on reasonable request.

## Abbreviations

BCG: Bacillus Calmette-Guerin Vaccine
CI: Confidence interval
CMR: Crude mortality rate
DRC: Democratic Republic of the Congo
ERB: Ethical Review Board
GAM: Global acute malnutrition
iCCM: Integrated community case management
IQR: Interquartile range
MAM: Moderate acute malnutrition
MCV: Measles containing vaccine
MoH: Ministry of Health
MSF: Médecins sans Frontières
MUAC: Mid upper arm circumference
OPV: Oral polio vaccine
PHC: Primary health centre
PHU: Primary health unit (in the study area these are health posts and community sites; they provide more basic care than the PHC)
PPS: Probability proportional to size
PSU: Primary sampling unit
SAM: Severe acute malnutrition
U5MR: Under-five mortality rate (mortality rate in children under five years of age)
WHO: World Health Organization
